# Overexpression of hsa-HLA-DRB1 may delay diabetic wound healing and angiogenesis by regulating miRNA_12118 and FLT-1

**DOI:** 10.1038/s41598-025-03906-8

**Published:** 2025-05-26

**Authors:** Chao Bai, Wenwen Yang, Jianghao Yan, Guangwei Qi, Liuyu Yang, Qingrui Wu, Jieguang Peng, Jun Luo, Tao Liu

**Affiliations:** 1 Department of Vascular and Thyroid Surgery, The First Affiliated Hospital of Xinjiang Medical University, Urumqi, 830054 Xinjiang China; 2 Postdoctoral Research Center of Public Health and Preventive Medicine, Xinjiang Medical University, Urumqi, 830000 P.R. China; 3 Department of Clinical Nutrition, The First Affiliated Hospital of Xinjiang Medical University, Urumqi, 830054 Xinjiang China; 4 School of Public Health, Xinjiang Medical University, Urumqi, 830000 P.R. China; 5 School of Public Health, Xinjiang Medical University, No. 567 Shangde North Road, Shimogou District, Urumqi, 830017 Xinjiang China

**Keywords:** Diabetic foot (DF), Circular RNAs, hsa-HLA-DRB1, miRNA_12118, FLT-1, Diabetes complications, Biological physics

## Abstract

We investigated the role and mechanism of hsa-HLA-DRB1 in the development and progression of diabetic foot ulcers. High-throughput sequencing was performed on three normal foot trauma tissues and diabetic foot ulcer tissues. The circRNAs with significant differences were identified. The downstream miRNAs were predicted by miRanda and RNAhybrid databases, and the mRNAs were predicted by the TargetScan database. Validation was performed with CCK8, flow cytometry, trabecular scratch assay, tubule generation assay, Western blot, dual luciferase assay, and RT-qPCR. High-throughput sequencing identified 461 significantly different circRNAs, of which 260 were up-regulated and 201 down-regulated. Compared to normal tissue, hsa-HLA-DRB1 was highly expressed in diabetic foot ulcers. The hsa-HLA-DRB1/miRNA_12118/FLT-1 axis was constructed. In vitro, we found that HLA-DRB1 overexpression inhibited cell viability, wound healing, and tubule formation, promoted apoptosis, and enhanced FLT-1 expression in HUVECs. The upregulation of hsa-HLA-DRB1 may promote diabetic foot development The upregulation of hsa - HLA - DRB1 may inhibit the biological function of endothelial cells by targeting miRNA_12118 and acting on FLT − 1. by targeting miRNA_12118 and acting on FLT-1. Therefore, our study highlights the key role of the hsa-HLA-DRB1/miRNA_12118/FLT-1 axis in diabetic foot trauma.

**Supplementary Information:**

The online version contains supplementary material available at 10.1038/s41598-025-03906-8.

## Introduction

Diabetes is a chronic metabolic disease with a rapidly increasing incidence in China and globally. As early as 2011, China became the country with the highest number of diabetes worldwide. Approximately 50% of individuals with diabetes are unaware of their condition^1,2^, leading to an earlier occurrence of associated complications, greatly affecting the quality of life and long-term prognosis of patients^3^.

Vascular damage, along with varying degrees of neurological impairment in the lower limbs of diabetic patients, ultimately results in the development of diabetic foot. The incidence of foot ulcers in diabetic patients is alarmingly high, estimated at 25%^4^. Consequently, this group of patients invariably experiences extended hospital stays, complicated and challenging treatment, expensive medical care, and a poor prognosis^5,6^. Shockingly, it is estimated that every 30 s worldwide, a diabetic amputation occurs due to trauma^4^. In diabetic patients, neuropathic lesions and inadequate tissue perfusion due to both macrovascular and microvascular impairments diminish the protective factors of the lower extremity, precipitating ulceration and gangrene, which subsequently increase the risk of amputation and mortality^7,8^.

Under high glucose conditions, several factors (such as endothelial cell inflammation and oxidative stress) can disrupt the balance between vasoconstrictor and diastolic functions and increase inflammation^9^. In turn, the expression of chemokines and adhesion molecules leads to endothelial dysfunction, reduced vasodilatation, and increased constriction, thereby promoting macrophage adhesion and migration, and exacerbating the development of atherosclerosis^10^. The development and progression of small atherosclerotic lesions, particularly in the terminal arteries, are widely believed to be shaped by inflammatory mechanisms^11,12^. This process is also considered to be the foundation for diabetic foot development.

Circular RNAs (circRNAs) are a class of novel non-coding RNAs characterized by their circular structure, which are found in eukaryotes. They exhibit high stability, conservation, and tissue specificity, which make them potential biological markers. They play important roles in various biological processes such as cell proliferation, apoptosis, metabolism, and inflammation^13^. Additionally, circRNAs have been found to regulate downstream microRNAs (miRNAs) and contribute to the occurrence and development of diseases. Specifically, circRNAs have been strongly associated with prostate cancer^14^, metastatic liver cancer^15^, breast cancer^16^, and lung cancer^17^. Moreover, preliminary studies suggest that circRNAs are implicated in both normal trauma^18^ and diabetic trauma^19^. Nevertheless, there is a scarcity of research exploring the association between circRNAs and diabetic foot or diabetic foot trauma.

Here, we explored the effects and mechanism of human leukocyte antigen DRB1 (hsa-HLA-DRB1) in diabetic foot ulcers.The functional characteristics of hsa-HLA-DRB1 as an “immune response switch” can influence autoimmune pathological processes through single-gene pleiotropy^20^. Meanwhile, prior studies have demonstrated that the HLA-DRB1 allele frequency in patients with type 2 diabetes mellitus is significantly higher than in control groups^21^. Diabetic foot tissue and tissue from normal foot trauma were collected. High-throughput assays were conducted to screen for significantly different circRNAs. The target relationships between circRNA, miRNA, and mRNA were analyzed by the miRanda, RNAhybrid, and TargetScan databases. Furthermore, in vitro validation was performed. The findings may provide a theoretical basis for understanding the pathogenesis of diabetic foot.

## Materials and methods

### Sample collection

A total of three cases of diabetic foot wound tissues were collected from the Department of Vascular and Thyroid Surgery, the First Affiliated Hospital of Xinjiang Medical University during wound debridement or dressing change.A total of three foot wound tissue samples from diabetic foot patients were collected from the Department of Vascular and Thyroid Surgery at the First Affiliated Hospital of Xinjiang Medical University. These samples were collected during debridement or dressing changes.Patients with diabetic foot were selected according to the following inclusion criteria: >18 years of age; history of type 2 diabetes mellitus; Wagner 3; 0.5 < ABI < 0.9; presence of an ulcer > 1 cm2 in the foot.Exclusion criteria were as follows: systemic infections; malignancies; charcot arthropathy.Wagner 3-grade patients have relatively consistent infection severity, extent of tissue damage, and clinical intervention requirements, which can reduce the interference of sample heterogeneity on sequencing analysis.Moderate ischemia (ABI 0.5–0.9) is a typical feature of DFU, retaining the impact of vascular lesions on wound healing while not reaching an irreversible terminal stage. This makes it more suitable for studying the relationship between ischemia and microbial/gene expression.Ulcers with larger areas are usually of longer duration (> 2 weeks), with more complex microbial colonization (such as biofilm formation.Exclude patients with severe complications to avoid exacerbating their conditions due to the study. and gene expression patterns that are closer to the chronic pathological state of DFU, avoiding the interference of acute wounds (such as new trauma).)

Three foot trauma tissues were collected during wound debridement or dressing change from individuals with normal open foot trauma from the Department of Emergency Medicine.Inclusion criteria for patients with foot trauma were as follows: age > 18.Exclusion criteria were as follows: history of diabetes mellitus; systemic infections; malignancies; charcot arthropathy.Ensure that the control group has no metabolic diseases or foot injuries, so as to clarify that the differences arise from diabetic foot itself rather than other factors.

Written consent was obtained from all participants. This study was carried out in accordance with Declaration of Helsinki and approved by the Ethics Committee of the First Affiliated Hospital of Xinjiang Medical University (Approval No. K201909-04).

### High-throughput sequencing

RNA-seq assays were conducted by Wuhan Ruixing Biotechnology Co., Ltd (http://www.rxbio.cc). Each sample underwent treatment with RQ1 DNase (M6101, Promega, USA) to eliminate DNA from total RNAs. Ribosomal RNAs were depleted using the Ribo-off™ rRNA depletion kit (N406, Vazyme, China). VAHTS^®^ Universal V8 RNA-seq Library Prep Kit for Illumina (NR605, Vazyme, China) was employed for RNA-seq library preparation. Fragmented RNAs were converted into double-stranded cDNA, followed by end repair, A tailing, and ligation to VAHTS RNA Multiplex Oligos Set 1 for Illumina (N323, Vazyme, China). The ligated products were then amplified, purified, quantified, and stored at −80 °C prior to sequencing. The strand marked with dUTP (the 2nd cDNA strand) was not amplified, allowing for strand-specific sequencing. High-throughput sequencing was carried out using the Illumina Novaseq 6000 system with the PE150 model.

### Sequencing data analysis

#### Genome mapping

Before circRNA identification, it is necessary to compare the effective sequencing data (clean reads) to the reference genome. According to the requirements of various circRNA identification tools, we employed three mapping software for comparison: HISAT2 (Findcirc), STAR (circrna_finder, circexplorer 2), and BWA (CIRI2).

#### CircRNA prediction

The identification of circRNA depends on the “back spliced reads”. We employed four commonly utilized circRNA prediction tools, namely Find_circ, CircRNA_finder, CIRCexplorer2, and CIRI2, to predict circRNAs individually. Subsequently, we filtered the circRNAs predicted by at least two tools to identify the final set of candidate circRNAs for downstream analysis.

#### CircRNA annotation

Based on the genome annotation, we first analyzed the splice sites of circRNA. Next, we determined the type and gene of the circRNA. We combined the annotation information from five known circRNA databases: circAltas, circBase, circRNADb, deepbase2, and circpedia2. Finally, we annotated them based on the ID of the candidate circRNA.

#### CircRNA sequence extraction

For identification of candidate circRNAs, we utilized the R-package FcircSEC (full-length circRNA sequence extraction and classification) to extract circular RNA sequences based on gene annotation and the output of any circRNA prediction tool.

#### CircRNA expression

SRPBM (Spliced Reads Per Billion Mapped Reads) is a standardized measure for circRNA expression and quantifies the number of back-spliced reads for each circRNA in every one billion reads. The formula to calculate SRPBM for a specific circRNA was presented as SRPBM (circRNA) = (back-spliced reads for circRNA) * 1,000,000,000/(total number of mapped reads).

### Functional enrichment analysis

Gene Ontology (GO) and KEGG pathway analyses were conducted using KOBAS 2.0 server. To determine the significance of the enrichment, a hypergeometric test and the Benjamini-Hochberg FDR controlling procedure were employed.

### Construction of the circRNA-miRNA-mRNA network

We identified the top 20 differentially expressed circRNAs (based on p-value) and excluded circRNAs that were only expressed in one sample. Next, we predicted the target miRNAs for these circRNAs using miRanda software (https://anaconda.org/bioconda/miranda), considering only those with a miRanda score greater than 150. Then, we predicted the target mRNA of these miRNAs by utilizing the intersection of the Targetscan (http://www.targetscan.org) and miRDB databases (http://mirdb.org). Subsequently, we intersected the identified mRNAs with the differentially expressed mRNAs in our project, distinguishing between upregulated and downregulated genes. Finally, we constructed the cirRNA-miRNA- mRNA network using the aforementioned results and visualized it using Cytoscape software.

### Cell culture and transfections

Human umbilical vein endothelial cells (HUVECs) serve as an endothelial cell model, capable of directly simulating the effects of the diabetic microenvironment (such as high glucose and advanced glycation end products, AGEs) on endothelial cells.Human Umbilical Vein Endothelial Cells (HUVECs) (Cat NO. DFSC-EC-01, Shanghai Zhong Qiao Xin Zhou Biotechnology Co., Ltd., China) were cultured at 37 °C with 5% CO2 in ECSM (ZQ-1304, Procell Life Science & Technology Co., Ltd., China) with 10% fetal bovine serum (Cat NO. 10091148, Gibco, China), 100 µg/mL streptomycin, 100 U/mL penicillin (Cat NO. SV30010, Hyclone, USA). To induce overexpression of hsa-HLA-DRB1, HUVECs were infected with a lentivirus carrying hsa-HLA-DRB1 (Genepharma, Suzhou, China) at an MOI (multiplicity of infection) of 35, for 72 h. The empty lentivirus was used as the negative control.

### CCK8 assay

The cell proliferation assay was performed using the Cell Counting Kit-8 (40203ES76, Yeasen, Shanghai, China). Infected HUVECs were seeded at a density of 10,000 cells per well in 96-well plates. After incubation at 37 °C and 5% CO2 for 24, 48, and 72 h, 10 µl of the CCK-8 solution was added to each well and further incubated for 3 h at 37 °C. The optical density of the cells was then measured at 450 nm using a Microplate Reader (ELX800, Biotek, USA). The following formula was used to calculate the cell proliferation rate: (OD450 of the experimental group - OD450 of blank control)/(OD450 of the negative control - OD450 of blank control) × 100%.

### Wound healing assay

HUVEC cells were seeded into six-well plates and cultured to 90% confluence. Then the cells was treated without serum for 4 h. A sterile 200 µL pipette tip was used to scratch straight lines in the cell layer to create wounds. Then, the cells were washed with PBS twice and cultured in ECSM (ZQ-1304, Procell Life Science & Technology Co., Ltd., China) with 10% fetal bovine serum (10091148, Gibco, China), 100 µg/mL streptomycin, 100 U/mL penicillin(SV30010, Hyclone, USA) at 37 °C. Wounds were observed at 0 and 21 h within the scrape lines, and representative points were marked and photographed at three individual fields.Complete wound closure was achieved in analyzed samples at ~ 21 h (measured via time-lapse imaging), while all scratches exhibited incomplete closure prior to this time point. Using the earliest closure time (21 h) as the experimental endpoint, wound closure rates were quantified across three biological replicates during this standardized observation period, ensuring comparative validity between groups. Then the wound closure was detected by inverted microscope (MF52-N, Mshot, China) at ×100 magnification. The area of wound gaps were measured using the ImageJ software. The cell mobility rate was calculated using the following formula: cell mobility rate (%) = (0 h Cell wound area − 21 h Cell wound area)/0 h Cell wound area × 100%.

### Tubule formation test

Infected HUVECs were seeded into 24-well plates pre-coated with matrigel (40183, Yeasen, Shanghai, China) at a volume of 250 µL per well, and incubated at 37 °C for 3 h. The images were observed under an inverted microscope (MF52-N, Mshot, China) at 100× magnification. Sprouting number and length were analyzed using ImageJ software.

### Flow cytometry

Cell apoptosis was detected using the Annexin V-APC/7-AAD apoptosis detection kit (40304ES60, Yeasen, Shanghai, China). After 72 h of infection, HUVECs were collected and treated with 5 µL Annexin V-APC in the dark at room temperature for 5 min, followed by incubation with 10 µL PI for 5 min. Subsequently, the samples were analyzed on a flow cytometer (FACSCanto, BD, USA) to determine the levels of cell apoptosis.

### Western blot

Infected HUVECs were lysed in ice-cold RIPA Buffer (PR20001, Proteintech, China) supplemented with a protease inhibitor cocktail (4693116001, Sigma, USA) for 30 min. The resulting samples were then loaded onto a 10% SDS-PAGE and subsequently transferred onto a 0.45 mm PVDF membrane (ISEQ00010, Millipore, USA). The PVDF membranes were blocked for 1 h at room temperature and incubated overnight at 4 °C with anti-FLT-1 (fms-related tyrosine kinase 1) (13687-1-AP, Proteintech, Wuhan, China) and anti-GAPDH (60004-1-Ig, Proteintech). Following this, the membranes were incubated with horseradish peroxidase-conjugated secondary antibodies (anti-rabbit, SA00001-2, or anti-mouse, AS003, ABclonal, Wuhan, China) for 45 min at room temperature. Finally, after color development with an enhanced ECL reagent (P0018 FM, Beyotime, Beijing, China), the membranes were visualized using chemiluminescence.

### Real-time quantitative PCR (RT-qPCR)

Total RNAs were extracted from infected HUVECs. The cDNA synthesis was conducted by standard procedures and RT-qPCR was performed on the QuantStudio5 with Hieff™ qPCR SYBR^®^ Green Master Mix (Low Rox Plus; YEASEN, China). The primer sequences were hum-*GAPDH*-F: GGTCGGAGTCAACGGATTTG, hum-*GAPDH*-R: GGAAGATGGTGATGGGATTTC; and, *FLT-1*-F: CATAGGTGCCTGAATCTTG, *FLT-1*-R: GCGGACAGTTAATAACAGAA. The relative level of each gene was normalized to GAPDH using the 2^−ΔΔCT^ method^22^.

### Statistical analysis

Statistical significance was assessed using a two-tailed Student’s t-test, Mann-Whitney U Test, Wilcoxon Matched Pairs Signed Rank Test, or two-way repeated-measures ANOVA. A p-value less than 0.05 was considered statistically significant.

## Results

### High-throughput sequencing results

**Fig. 1:**
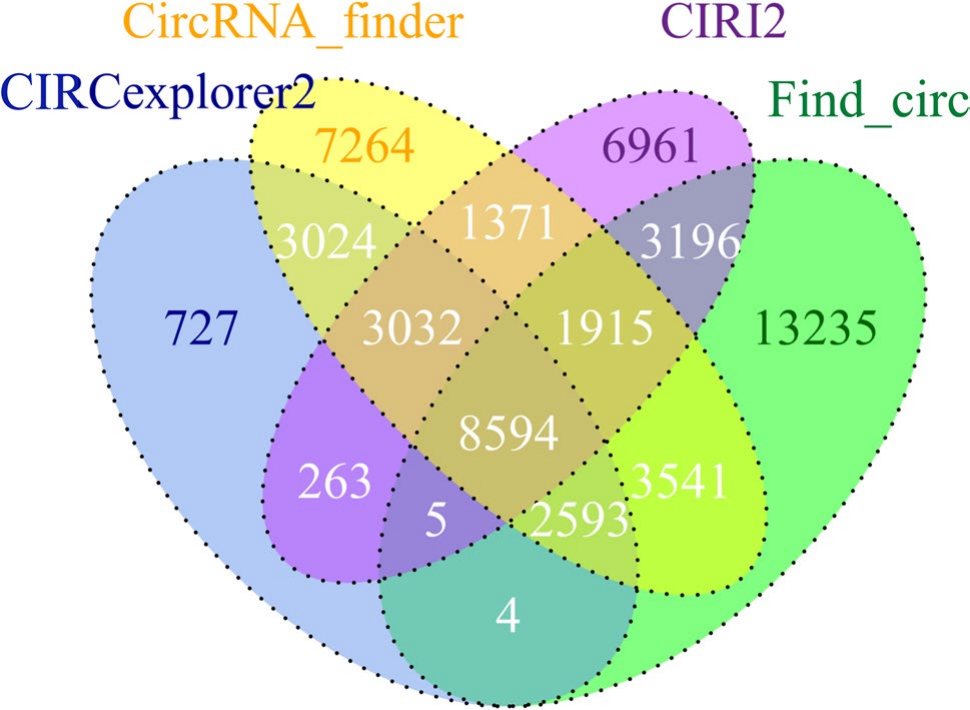
Venn diagram illustrating the screening of circRNAs.

High-quality transcriptome data were obtained after Novaseq 6000 sequencing. Four circRNA prediction tools (Find_circ, CircRNA_finder, CIRCexplorer2, and CIRI2) were used to predict circRNAs. A total of 55,725 circRNAs were predicted, including 18,242 by CIRCexplorer2, 31,334 by circRNA_finder, 25,337 by CIRI2, and 33,083 by Findcirc (Fig. 1). The circRNAs predicted by at least 2 tools were determined as final candidate circRNAs and subjected to subsequent analysis.

### Identification of differentially expressed circrnas

**Fig. 2:**
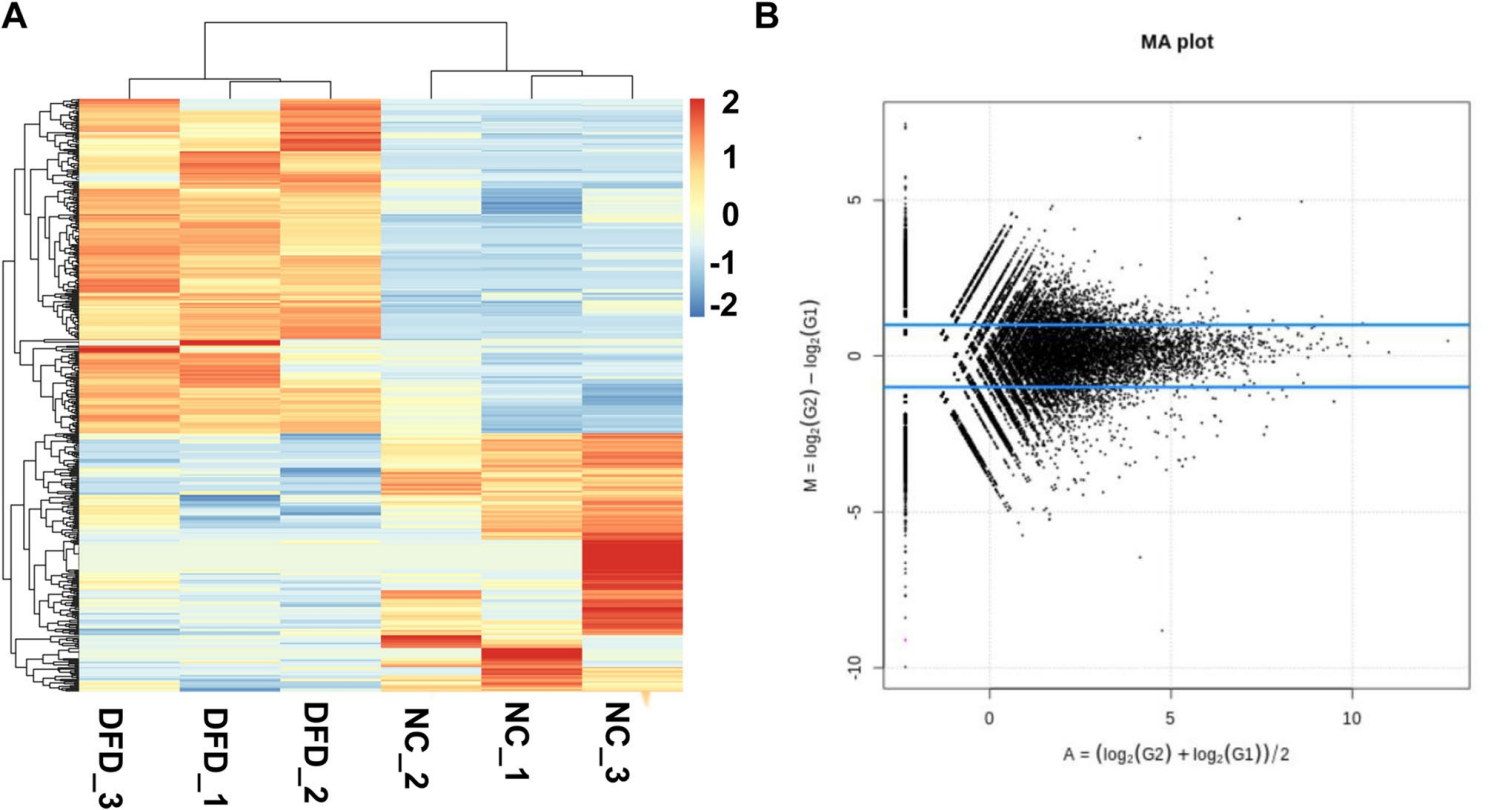
Screening of differentially expressed circRNAs. **(A)** Heat map. **(B)** MA plot. NC, normal control tissue; DFD, diabetic foot tissue.

The differentially expressed circRNAs between normal tissue and diabetic foot tissue were screened. A total of 27,538 circRNAs were screened, of which 461 were significantly differentially expressed, including 260 up-regulated circRNAs and 201 down-regulated circRNAs, as revealed by the heat map (Fig. 2A) and MA plot (Fig. 2B).

### Gene ontology (GO) enrichment analysis of host genes of differentially expressed circrnas

**Fig. 3:**
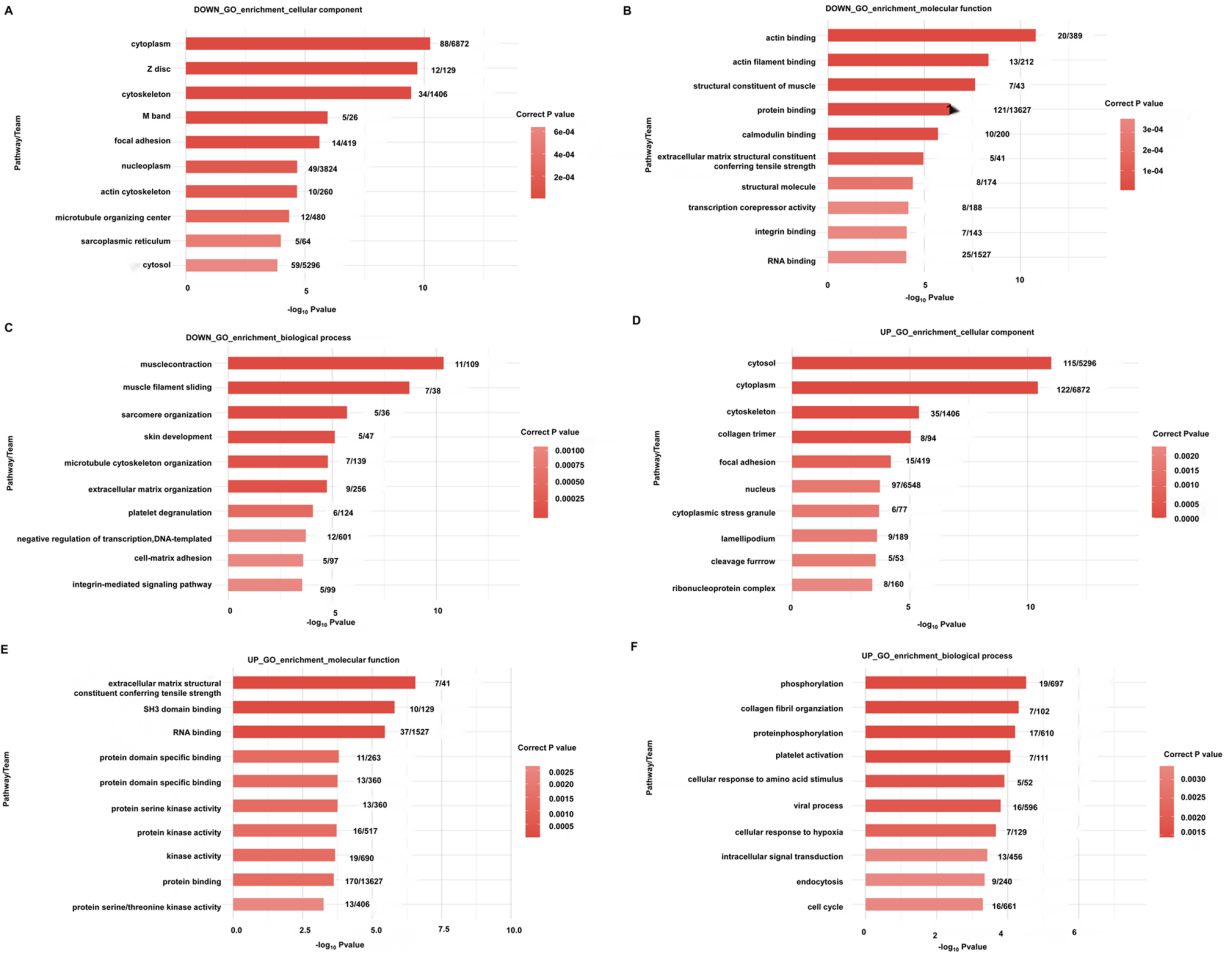
GO enrichment analysis. The top 10 enriched GO terms are displayed. **(A)** The enriched GO terms of host genes of down-regulated circRNAs in Cellular Component. **(B)** The enriched GO terms of host genes of down-regulated circRNAs in Molecular Function. **(C)** The enriched GO terms of host genes of down-regulated circRNAs in Biological Process. **(D)** The enriched GO terms of host genes of up-regulated circRNAs in Cellular Component. **(E)** The enriched GO terms of host genes of up-regulated circRNAs in Molecular Function. **(F)** The enriched GO terms of host genes of up-regulated circRNAs in Biological Process.

To denote gene function, the GO enrichment analyses of host genes that differentially expressed circRNAs were performed. The top 10 enriched GO terms were displayed in Fig. 3. For the down-regulated genes, the top 10 GO terms enriched in cellular component included extracellular region, collagen-containing extracellular matrix, lipid droplet, extracellular matrix, extracellular space, receptor complex, basement membrane, extracellular exosome, chromatin, and integral component of plasma membrane (Fig. 3A); those in molecular function included oxidoreductase activity, acting on the aldehyde or oxo group of donors, NAD or NADP as acceptor; retinol dehydrogenase activity; extracellular matrix structural constituent; heparin binding; oxidoreductase activity; DNA-binding transcription activator activity, RNA polymerase II-specific; sequence-specific double-stranded DNA binding; signaling receptor binding; DNA-binding transcription factor activity; and, protein homodimerization activity (Fig. 3B); and, those in biological process included retinol metabolic process; cellular response to retinoic acid; retinoid metabolic process; cell adhesion; oxidation − reduction process; animal organ morphogenesis; ossification; glucose homeostasis; lipid metabolic process; and response to bacterium (Fig. 3C).

For the up-regulated genes, the top 10 GO terms enriched in cellular component included extracellular region; tertiary granule membrane; plasma membrane; secretory granule membrane; extracellular space; integral component of plasma membrane; extracellular matrix; integral component of membrane; membrane; and neuronal cell body (Fig. 3D); those in molecular function included calcium − dependent protein binding; calcium ion binding; protein homodimerization activity; carbohydrate binding; actin binding; actin filament binding; protein binding; G protein − coupled receptor activity; zinc ion binding; and peptidase activity (Fig. 3E); and, those in biological process included neutrophil degranulation; chemotaxis; defense response to bacterium; inflammatory response; signal transduction; immune system process; cell − cell signaling; cytokine − mediated signaling pathway; neutrophil chemotaxis, and innate immune response (Fig. 3F).

### KEGG pathway enrichment analysis of the host genes of differentially expressed circrnas

**Fig. 4:**
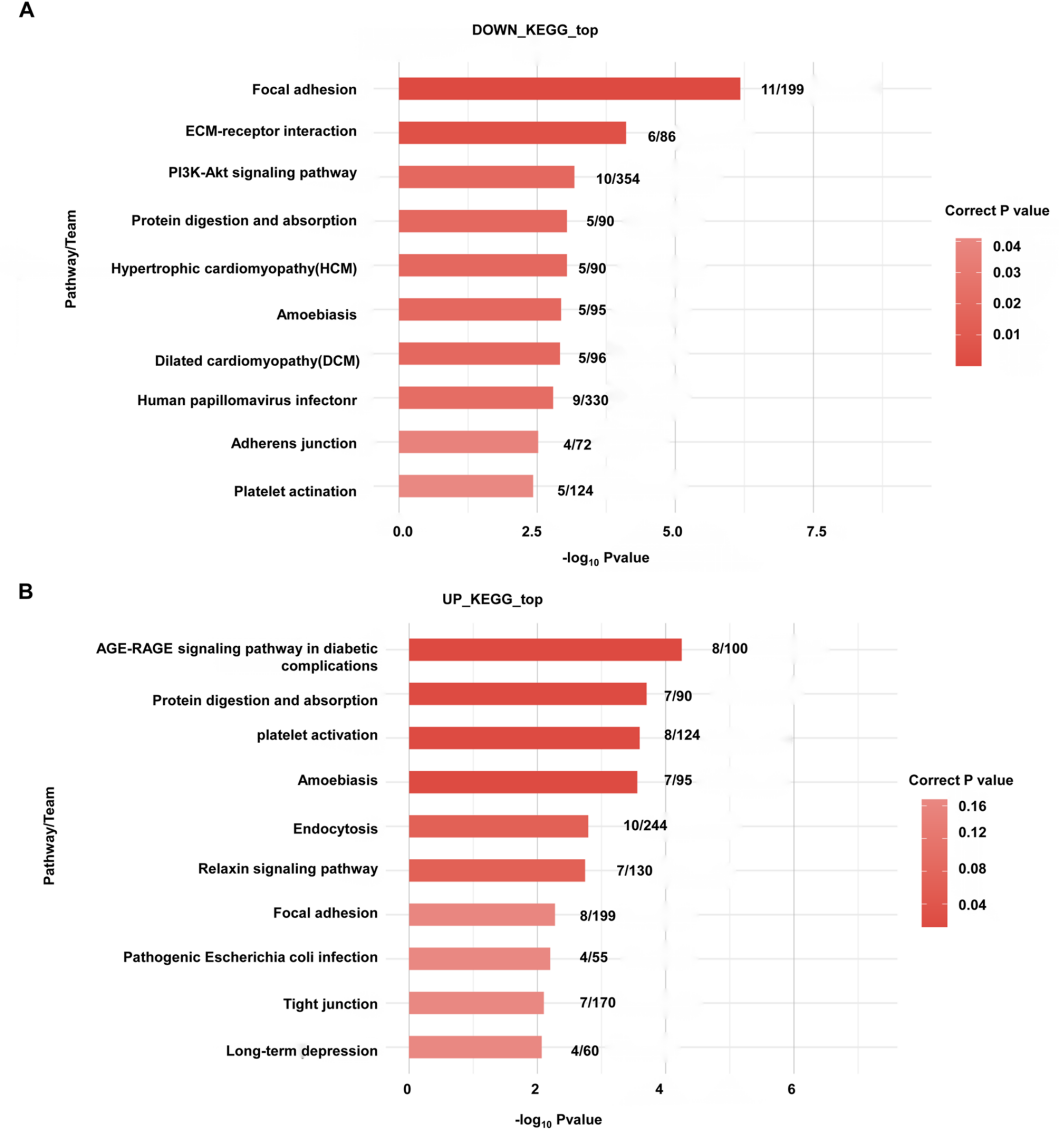
KEGG pathway enrichment analysis. The top 10 enriched KEGG pathways are displayed. **(A)** The enriched KEGG pathways of host genes of down-regulated circRNAs. **(B)** The enriched KEGG pathways of host genes of up-regulated circRNAs.

The host genes of differentially expressed circRNAs were also subjected to KEGG pathway enrichment analysis (Fig. 4). The top 10 enriched KEGG pathways were displayed. As shown in Fig. 4A, the top 10 enriched KEGG pathways for the down-regulated genes were PPAR signaling pathway; Retinol metabolism; Regulation of lipolysis in adipocytes; Malaria; Cocaine addiction; ECM − receptor interaction; Alanine, aspartate and glutamate metabolism; Wnt signaling pathway; Amphetamine addiction; and Adipocytokine signaling pathway. For the up-regulated genes, the top 10 enriched KEGG pathways included IL − 17 signaling pathway; Staphylococcus aureus infection; Transcriptional misregulation in cancer; Arginine biosynthesis; Osteoclast differentiation; Galactose metabolism; Starch and sucrose metabolism; Rheumatoid arthritis; Inflammatory mediator regulation of TRP channels; and, Viral protein interaction with cytokine and cytokine receptor (Fig. 4B).

### Construction of the circRNA-miRNA-mRNA network

**Fig. 5:**
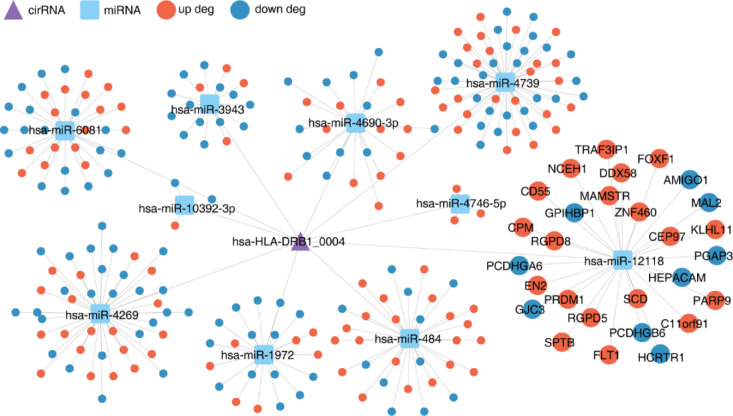
The circRNA-miRNA-mRNA network.

We evaluated the target relationships between circRNA, miRNA, and mRNA by using the miRanda, RNAhybrid, and TargetScan databases. The circRNA-miRNA-mRNA network was constructed (Fig. 5).

### Role of hsa-HLA-DRB1/miRNA_12118/FLT-1 in diabetic foot

**Fig. 6:**
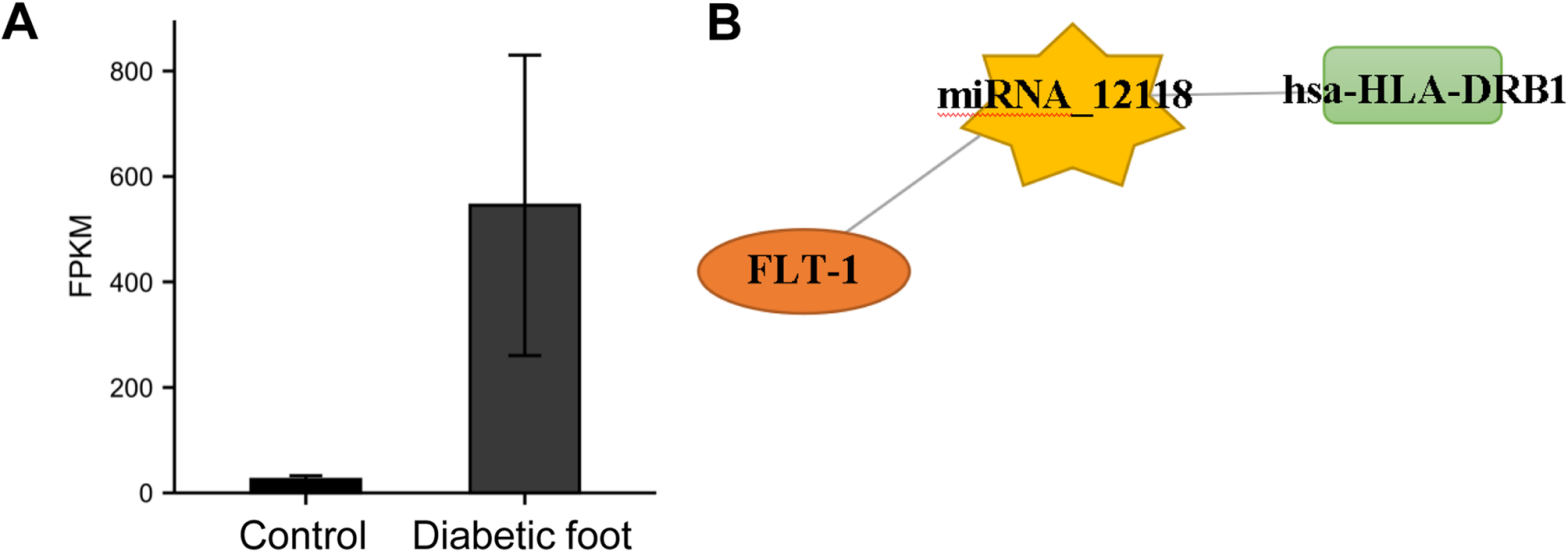
Analysis of hsa-HLA-DRB1/miRNA_12118/FLT-1 axis. **(A)** Comparison of hsa-HLA-DRB1 expression in diabetic foot and normal foot traumatic tissue. **(B)** The hsa-HLA-DRB1/miRNA_12118/FLT-1 axis.

The expression of hsa-HLA-DRB1 was validated in three cases of diabetic foot trauma and three cases of normal trauma tissues by using RT-qPCR. As shown in Fig. 6A, there was a significant difference in hsa-HLA-DRB1 between the two groups. Furthermore, an extensive literature review has demonstrated the important roles of miRNA_12118 and FLT-1 in the inflammatory-mediated pathways involved in the development and progression of diabetic foot^23,24^. Therefore, the hsa-HLA-DRB1/miRNA_12118/FLT-1 may play a critical role in regulating the occurrence and development of diabetic foot through the inflammatory pathway (Fig. 6B).

### Overexpression of hsa-HLA-DRB1 inhibits cell viability

**Fig. 7:**
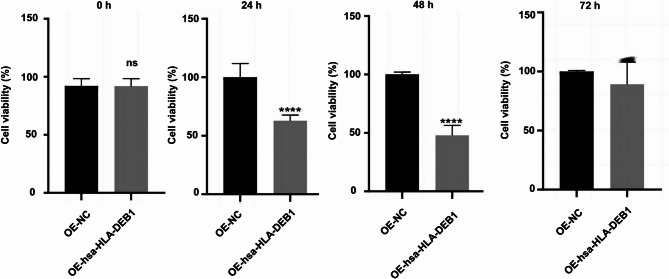
Cell viability after hsa-HLA-DRB1 overexpression. CCK8 assay detected cell viability after hsa-HLA-DRB1 overexpression for 0 h, 24 h, 48 h, and 72 h. Compared with OE-NC, ***P < 0.001.

The effect of hsa-HLA-DRB1 overexpression on the viability of HUVECs was determined by CCK8 assay. As displayed in Fig. 7, after infection for 24 h and 48 h, the cell viability of HUVECs with hsa-HLA-DRB1 overexpression was significantly lower than that of the control.

### Overexpression of hsa-HLA-DRB1 suppresses wound healing

**Fig. 8:**
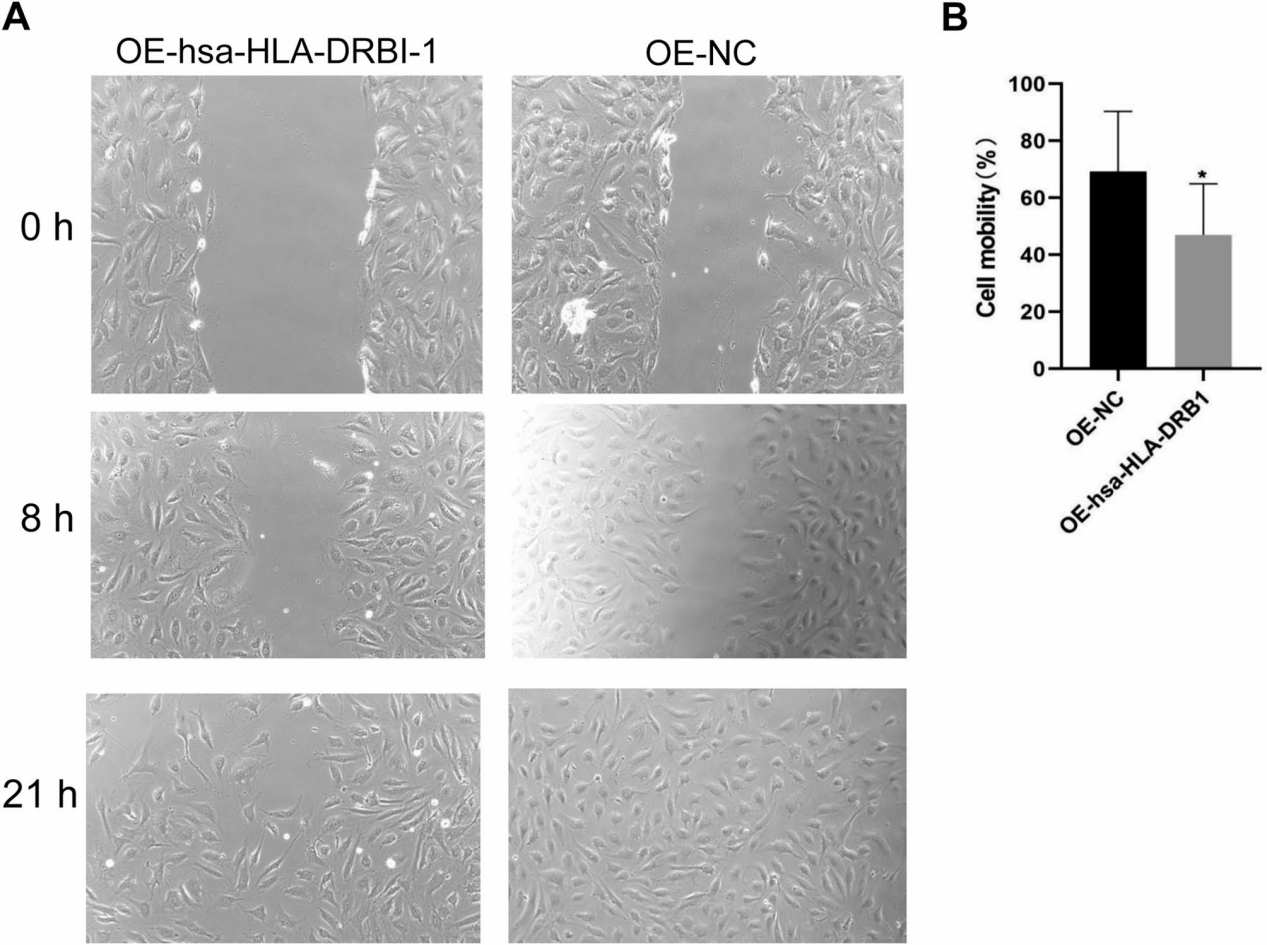
Analysis of wound healing after hsa-HLA-DRB1 overexpression. **(A)** Representative images of wound healing at 0 h, 8 h, and 21 h after scratching. **(B)** Comparison of cell mobility. Compared with OE-NC, *P < 0.05.

Wound healing assay evaluated the effect of hsa-HLA-DRB1 overexpression on cell mobility. Overexpression of hsa-HLA-DRB1 in HUVEs cells significantly decreased cell mobility compared to the control group after 21 h of scratching (Fig. 8A and B). Thus, the hsa-HLA-DRB1 may have an inhibitory effect on cell wound healing.

### Overexpression of hsa-HLA-DRB1 inhibits tubule formation

**Fig. 9:**
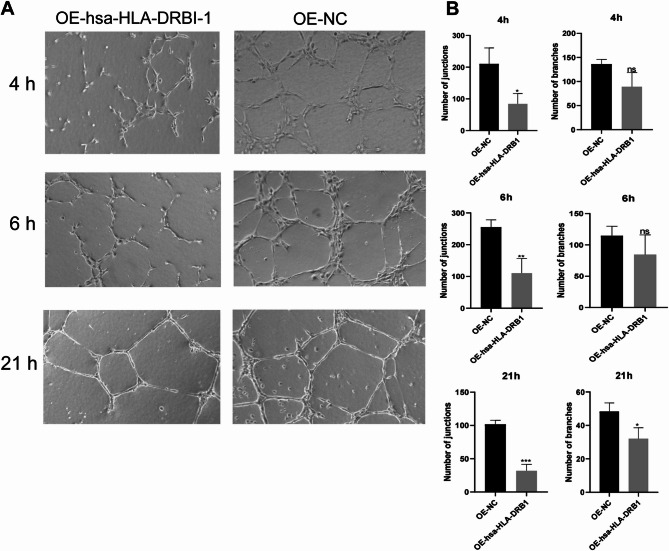
Analysis of tubule formation after hsa-HLA-DRB1 overexpression. **(A)** Representative images of tubule formation at 4 h, 6 h, and 21 h. **(B)** Comparison of the number of branches and junctions. Compared with OE-NC, *P < 0.05, **P < 0.01, ***P < 0.001. ns, not significant.

In HUVEC cells, overexpression of the hsa-HLA-DRB1 gene inhibited the number of branches and junctions at 4 h, 6 h, and 21 h compared with the control group (Fig. 9A and B). This indicates that overexpression of hsa-HLA-DRB1 may inhibit angiogenesis.

### Overexpression of hsa-HLA-DRB1 promotes apoptosis

**Fig. 10:**
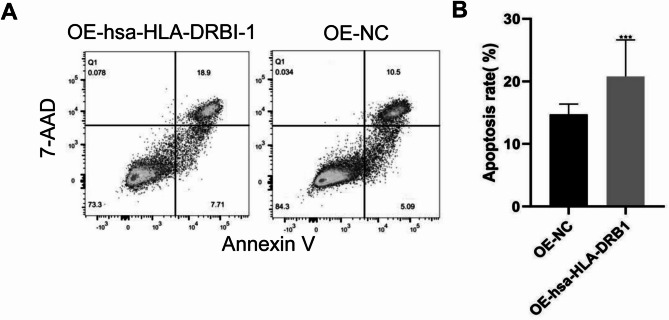
Analysis of apoptosis after hsa-HLA-DRB1 overexpression. **(A)** Representative flow cytometry results. **(B)** Comparison of apoptosis rates. Compared with OE-NC, ***P < 0.001.

We performed flow cytometry to detect cell apoptosis. The representative flow cytometry results are shown in Fig. 10A. Statistically, HUVEC cells with overexpression of OE-hsa-HLA-DRB1 had a significantly higher apoptosis rate (Fig. 10B), indicating the promotive effect of hsa-HLA-DRB1 on apoptosis.

### Overexpression of hsa-HLA-DRB1 enhances FLT-1 expression

**Fig. 11:**
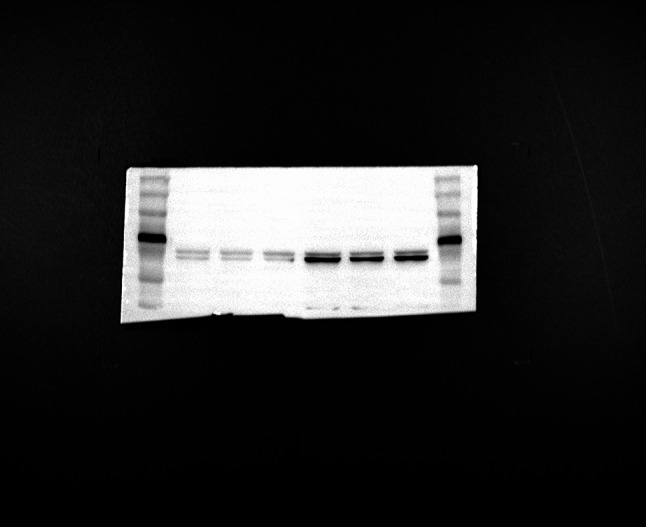
Analysis of FLT-1 after hsa-HLA-DRB1 overexpression. FLT-1 expression in HUVEC cells with overexpression of hsa-HLA-DRB1 was detected by Western blot and RT-qPCR. **(A)** Western blot results. **(B)** RT-qPCR. Compared with NC, ***P < 0.001.

FLT-1 was identified as one of the downstream mRNAs of hsa-HLA-DRB1. Thus, we validated FLT-1 expression in HUVEC cells with overexpression of hsa-HLA-DRB1 by Western blot and RT-qPCR. As shown in Fig. 11A and B, both the protein and mRNA levels of FLT-1 increased significantly in HUVECs overexpressing hsa-HLA-DRB1 compared to the control group. This suggests that hsa-HLA-DRB1 may up-regulate FLT-1 in HUVEC cells.

## Discussion

The onset and development of diabetic foot is an extremely complex process. Understanding the molecular mechanism underlying its occurrence can offer a theoretical foundation for future treatment strategies for diabetic foot. The circRNAs exhibit high specificity and stability, making them pivotal factors in numerous diseases. In this study, we screened 461 circRNAs using high-throughput detection in diabetic foot tissues and normal foot trauma tissues. Among them, 260 circRNAs were found to be up-regulated, while 201 were down-regulated. This study provides strong evidence suggesting that hsa-HLA-DRB1 and other circRNAs may play significant functional roles in the development of diabetic foot ulcers. Moreover, both high-throughput sequencing and validation experiments showed that hsa-HLA-DRB1 was highly expressed in diabetic foot tissue. In vitro validation further showed that hsa-HLA-DRB1 overexpression inhibited cell viability, wound healing, and tubule formation, promoted apoptosis, and enhanced FLT-1 expression. Of note, the hsa-HLA-DRB1/miRNA_12118/FLT-1 axis was identified, which may have a crucial role in the development of diabetic foot ulcers. Further studies are warranted to determine the biological functions of these diabetic foot ulcer-related circRNAs and whether they may be involved in the pathogenesis of chronic nonhealing wounds.

The functional role of circRNAs in human skin wound healing has been reported. For example, Wang et al.^25^ identified hsa_circ_0084443 as significantly differentially expressed in diabetic foot ulcer tissues compared to normal wound tissues using circRNA microarray analysis. By using Western blot, RT-qPCR, and fluorescence in situ hybridization techniques, cell migration, cell growth, and other assays, higher levels of hsa_circ_0084443 were detected in diabetic foot ulcer samples^25^. Zeng et al.^26^ retrieved gene expression profiles from the GEO database and divided the samples into control and diabetic foot ulcers. A competing endogenous RNA (ceRNA) network was constructed by performing GO and KEGG pathway enrichment analyses on the differentially expressed genes. Ultimately, the ceRNA network was constructed. The pathway analysis revealed that VEGF and T cell receptor signaling pathways were predominantly enriched in individuals with diabetic foot ulcers. They unveiled the potential association between important ceRNAs (JUNB, GATA3, hsa_circ_0049271, and hsa_circ_0074559) and infiltrating immune cells (CD8 + T cells and monocytes) with a diabetic foot ulcer.

To understand the mechanisms underlying the biological effects of hsa-HLA-DRB1, we identified the axis of hsa-HLA-DRB1/miRNA_12118/FLT-1, a pathway important for diabetic wound healing. FLT-1 is a receptor for VEGF. Abd El-Khalik et al.^27^ reported that sFlt-1, AOPPs, MDA, and TNF-α, as well as VEGF, were significantly elevated in diabetic patients, with the highest levels in patients with diabetic foot ulcers. Natrus et al.^23^ demonstrated that neutrophils were the primary producers of VEGF and played a role in stimulating FLT-1 expression. The presence of hyperglycemia disrupts the balance of angiogenic receptors and ligands (VEGF and FLT-1), potentially leading to chronic inflammation, hypoxia, and inhibition of burn wound healing. This imbalance may also impede the regeneration of damaged tissue in individuals with diabetes. In a correlation study, Bayat et al.^28^ examined flap transplantation in both healthy rats and rats with T1DM (type 1 diabetes). The study findings suggest that T1DM rats exhibited elevated levels of both intact and degranulated mast cells, and there was a positive correlation between mast cell degranulation and FLT-1 expression. This correlation may contribute to poor angiogenesis and reduced vascular function, ultimately leading to decreased flap survival.

The results of this study, along with related literature^27,29^, suggest that FLT-1 plays a role in the angiogenesis process by mediating inflammation. Preliminary evidence suggests that the hsa-HLA-DRB1/miRNA_12118/FLT-1 pathway potentially regulates wound healing through an inflammatory pathway in a high-glucose environment.

Several limitations of the current study should be noted. The majority of the data in our study were obtained from cellular experiments.Human umbilical vein endothelial cells (HUVECs) are a single cell type and cannot fully simulate the complex cellular interactions present in diabetic foot ulcer (DFU) wounds. Additionally, endothelial cells derived from umbilical veins may have epigenetic differences compared to the diseased endothelium in diabetic patients. As a result of the absence of animal experiments, we cannot directly claim that our findings contribute to the understanding of diabetic foot. Consequently, further in vitro studies using animal models are necessary to investigate the precise involvement of the hsa-HLA-DRB1/miRNA_12118/FLT-1 axis in the diabetic foot.

In conclusion, the current data suggest that the hsa-HLA-DRB1 may contribute to the development of diabetic foot by targeting miRNA_12118 and acting on FLT-1. Thus, our study highlights the pivotal role of the hsa-HLA-DRB1/miRNA_12118/FLT-1 axis in regulating diabetic foot trauma. Moreover, our findings strongly support the functional and potential clinical significance of circRNAs in the healing of cutaneous wounds.Develop drugs targeting HLA-DRB1 or its downstream signaling pathways to reduce the release of inflammatory factors like TNF-α and IL-1β, improving the wound microenvironment. Combine these drugs with existing wound treatment technologies, tailoring treatment plans according to traditional wound cleaning and genetic test results. For example, enhance the use of anti-inflammatory dressings or growth factors in patients with high HLA-DRB1 expression to combat inflammation and promote healing. Further efforts are required to comprehend the precise role and potential molecular mechanisms of these circRNAs associated with diabetic foot ulcers. Such investigations will unveil novel and unexpected biological properties, leading to a better understanding of the pathophysiology of diabetic foot ulcers.

## Electronic supplementary material

Below is the link to the electronic supplementary material.

Supplementary Material 1

Supplementary Material 2

Supplementary Material 3

## Data Availability

All raw data from our manuscript have been successfully uploaded to the Gene Expression Omnibus (GEO) database. The GEO accession number assigned is GSE286165.
