# Supplementary figures and images for: Overexpression of hsa-HLA-DRB1 may delay diabetic wound healing and angiogenesis by regulating miRNA_12118 and FLT-1
Source: Sci Rep. 2025 May 26;15:18409. doi: 10.1038/s41598-025-03906-8 (PMC12106794; doi:10.1038/s41598-025-03906-8)

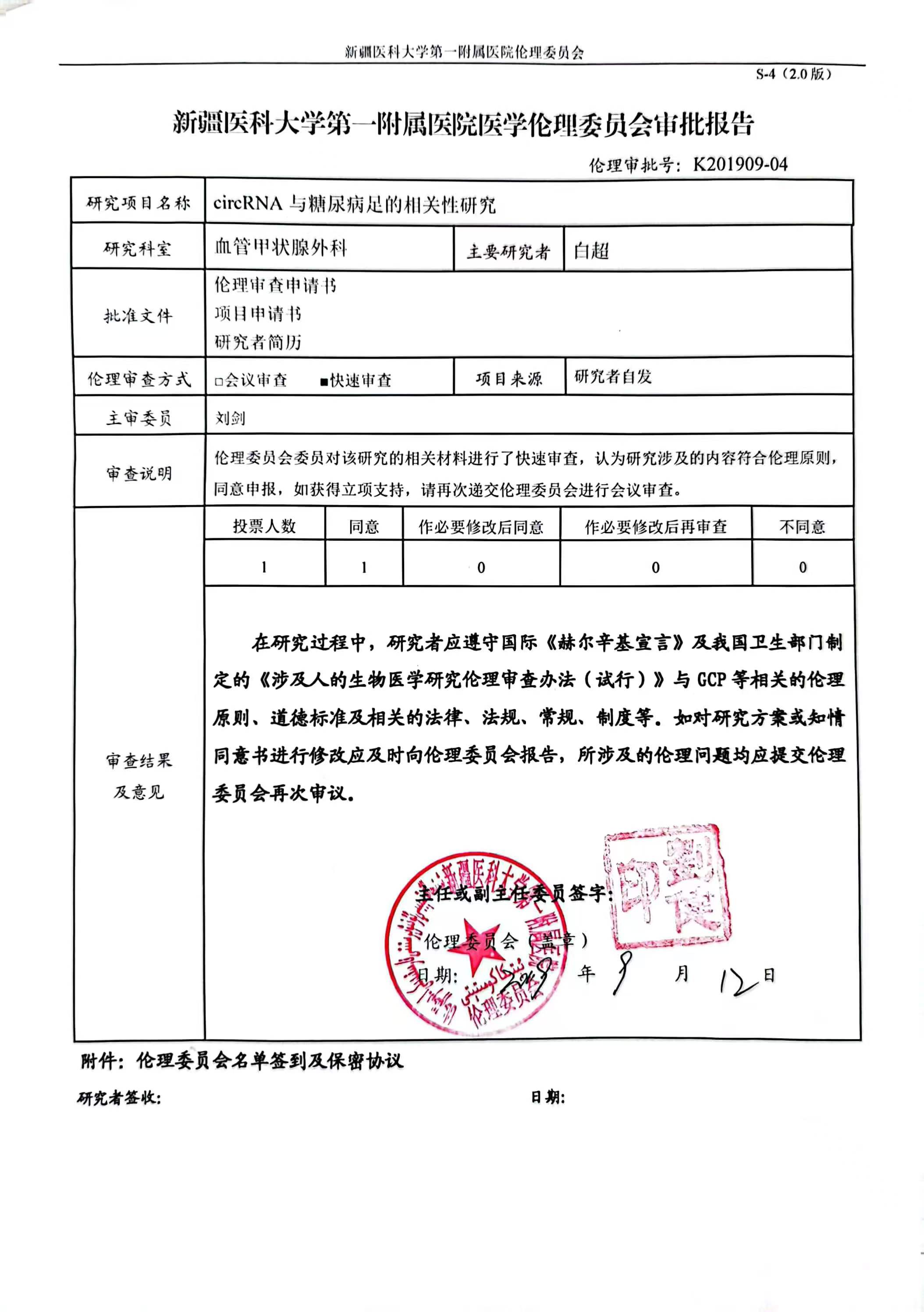

Supplement: Supplementary file 1 — Supplementary Material 1 [file 41598_2025_3906_MOESM1_ESM.jpg]

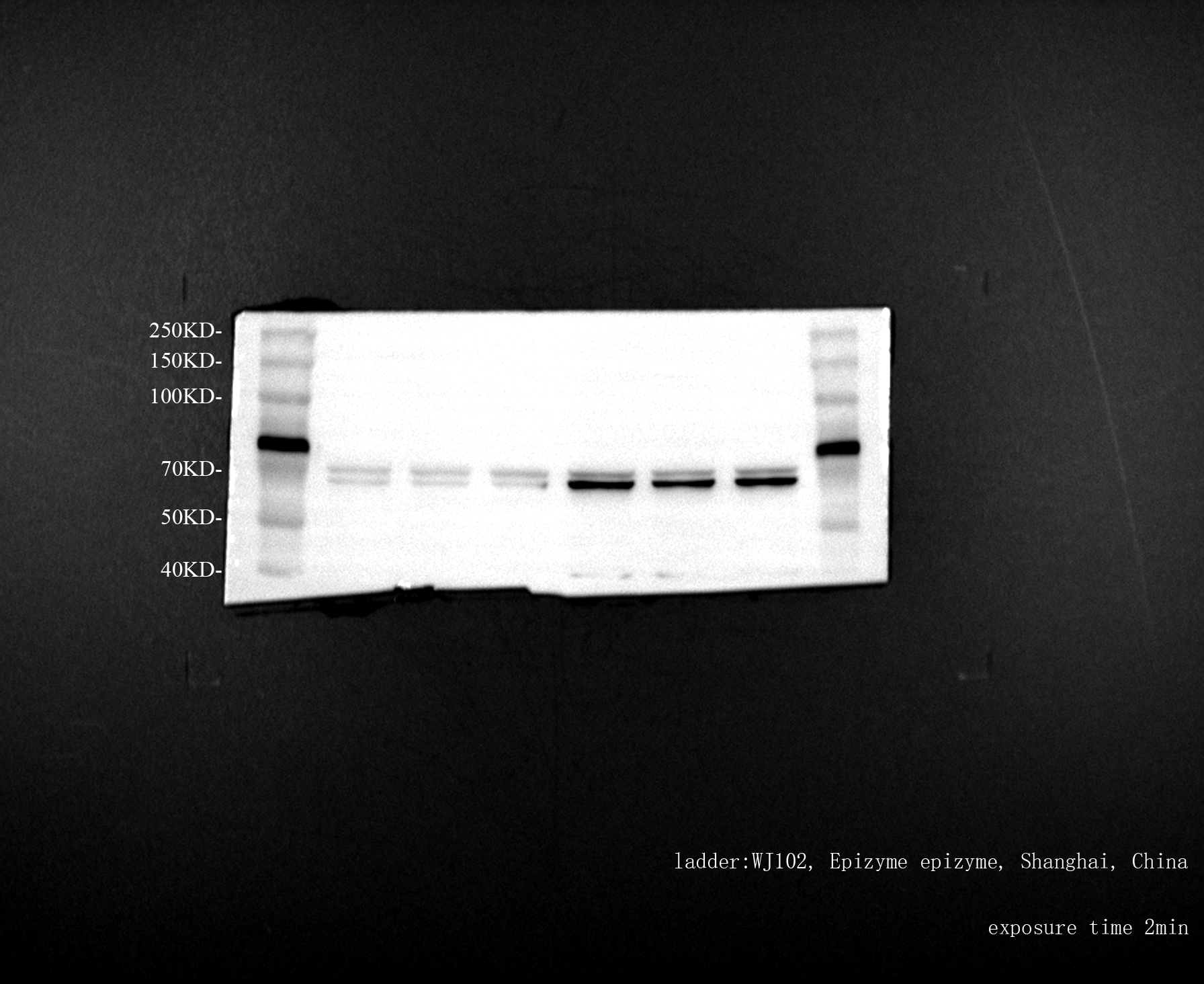

Supplement: Supplementary file 2 — Supplementary Material 2 [file 41598_2025_3906_MOESM2_ESM.tif]

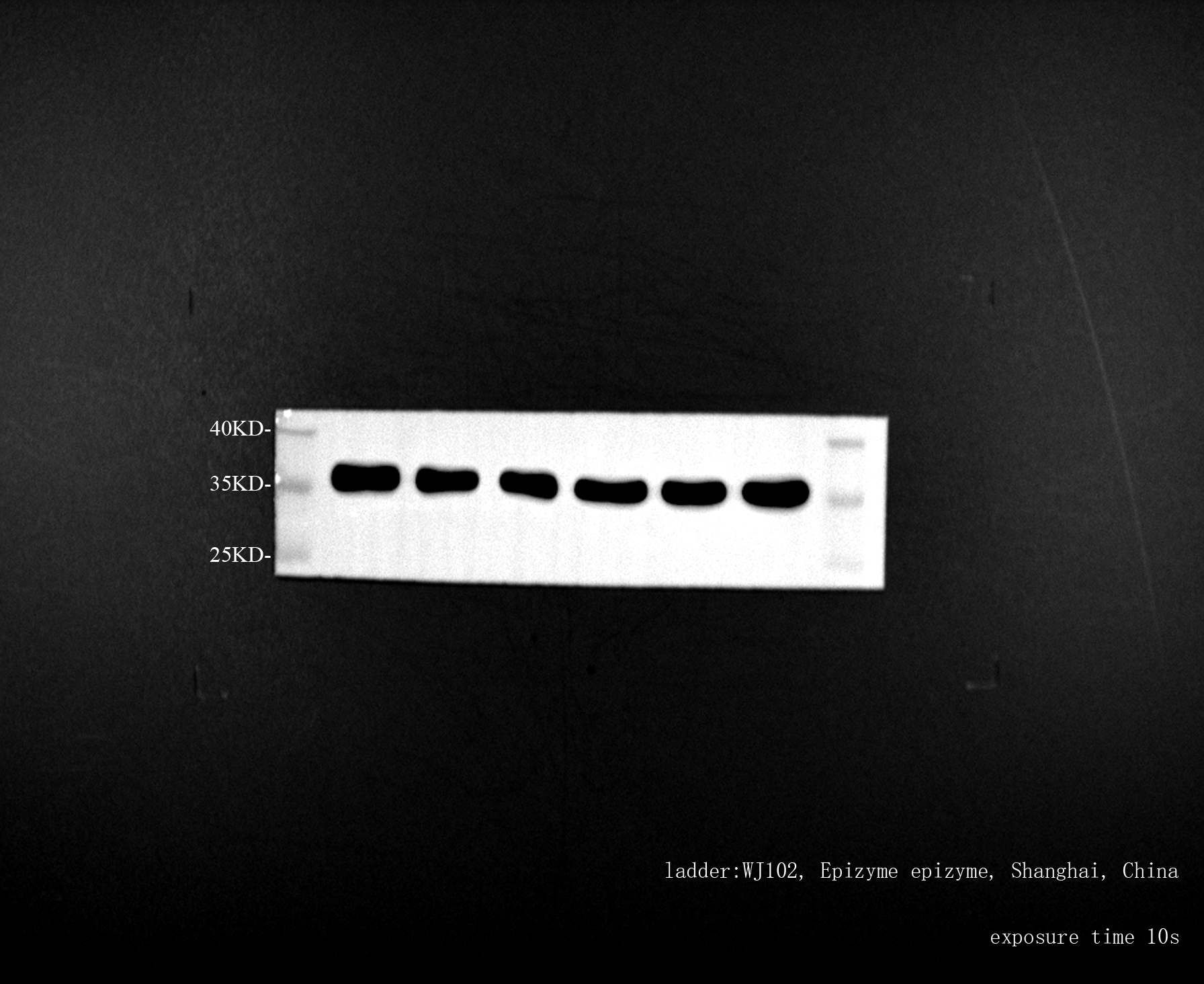

Supplement: Supplementary file 3 — Supplementary Material 3 [file 41598_2025_3906_MOESM3_ESM.tif]
